# Morphological and anatomical changes related to leg anomalies in *Tegenaria atrica*

**DOI:** 10.1007/s00435-015-0260-0

**Published:** 2015-02-27

**Authors:** Teresa Napiórkowska, Paweł Napiórkowski, Julita Templin

**Affiliations:** 1 Department of Invertebrate Zoology, Faculty of Biology and Environmental Protection, Nicolaus Copernicus University, 87-100 Toruń, Lwowska 1, Poland; 2 Department of Hydrobiology, Faculty of Biology and Environmental Protection, Nicolaus Copernicus University, Toruń, Lwowska 1, Poland

**Keywords:** Malformations, Central nervous system, Teratogenic factor, Spider

## Abstract

A range of leg anomalies was detected in embryos of the *Tegenaria atrica* spiders exposed to alternating temperatures of 14 and 32 °C. Multiple anomalies were observed in 13 individuals. This study is based on five individuals: two individuals affected by oligomely combined, respectively, with heterosymely and polymely, one affected by polymely with heterosymely, one by complicated polymely (accompanied by the reduction in length and malformations of the distal parts of the legs), and one individual with pure polymely. Changes in the central nervous system of these five individuals were described in detail on the basis of histological sections. The changes were mainly related to the number of neuromeres. Individuals affected by polymely had additional ganglia corresponding to the number of additional appendages, whereas the absence of a leg (oligomely) was associated with the absence of a ganglion. Histological analysis showed the fusion of ganglia in the three polymelic specimens, even though additional appendages were not fused.

## Introduction

The literature provides many descriptions of invertebrates (including arachnids) with deformities of the whole body or single body parts (e.g. Anderson 1961; Mikulska and Martinek 1964; Yaginuma and Arita 1966; Williams 1971). These anomalies are recorded mostly during faunistic studies, and as their causes are not known, they are referred to as idiopathic. Besides spontaneous anomalies of unknown aetiology, there are also those which can be induced in laboratory using chemical, physical, or mechanical factors including temperature. Holm (1940) was the first to put forward a hypothesis about a teratogenic effect of temperature in spiders. In a later period, Juberthie (1962, 1963a, b, 1968) studied the impact of elevated temperature on the embryonic development of harvestmen (Opilionida). He discovered that increased temperature caused miscellaneous leg malformations, i.e. oligomely (reduction in the number of legs), polymely (presence of an additional appendage or appendages), heterosymely (fusion of legs located next to each other, on the same side of the body) or symely (fusion of legs from the same pair). Similar malformations were observed by Mikulska (1973) who applied elevated temperature to embryos of the spider *Argyroneta aquatica* (Clerck). Further laboratory experiments involving embryos of the *Tegenaria atrica* C. L. Koch showed that temperatures higher and lower than optimum, applied alternately during the incubation of embryos, had a stronger teratogenic effect than supraoptimal temperature only (Jacuński 1984).

Obviously, leg malformations are reflected in the morphological and anatomical structure of spiders. Internal deformities are found mainly in the digestive system and the central nervous system (Jacuński 1983; Jacuński et al. 2002, 2005; Napiórkowska et al. 2006, 2010, 2013). The central nervous system of *Poecilotheria* and *Argiope aurantia* (Lucas) was described in detail by Babu (1965, 1969, 1975). It was also investigated by Babu and Barth (1984), Wegerhoff and Breidbach (1995), Barth (2002), Hwang and Moon (2003), Hill (2006), Park and Moon (2013), and Park et al. (2013). The central nervous system of spiders can be described as a highly condensed organ consisting of two parts: the supraoesophageal ganglion (brain) and the fused suboesophageal ganglia, both located in the prosoma. In the supraoesophageal ganglion located in the anterior-dorsal part of the prosoma, there are the ganglia of chelicerae as well as optical and associative centres (Satija et al. 1970a, b, 1980; Strausfeld and Barth 1993; Strausfeld et al. 1993). The fused suboesophageal ganglia consist of the pedipalp ganglia, the four pairs of ganglia of walking appendages and the opisthosomal ganglia. The pedal ganglia are relatively large, separated from each other, and arranged in a large, star-like structure, reflecting the metamerism of the prosoma.

Some anomalies may affect the structure of the nervous system. Structural changes in the fused suboesophageal ganglia were previously recorded in different cases of oligomely (Jacuński et al. 2005) and heterosymely (Napiórkowska et al. 2013) of *T. atrica*. However, no information is available about defects of the nervous system of spiders affected by multiple anomalies. Before the experiment, we assumed that anomalies of this kind might affect not only the overall shape of the body but also the structure of the central nervous system (CNS). The aim of the experiment was to show the connection between body deformities and changes in the internal structures of *T. atrica*.

## Materials and methods

The study material consisted of individuals of *T. atrica* C. L. Koch, 1843. Embryos for teratological studies were obtained by breeding males and females caught alive in the natural environment near the cities of Toruń and Chełmża (Poland) in August and September each year from 2006 to 2012. The collection of 162 females and 47 males was reared in laboratory. Each sexually mature individual was kept in a ventilated, 250 cm^3^ glass container, in the optimal conditions for the species, i.e. at a temperature of 21–23 °C and relative humidity of ca. 70 % (Mikulska and Jacuński 1968; Jacuński et al. 1994). The spiders were fed twice a week with *Tenebrio molitor* larvae, and a water-soaked cotton ball was used to provide water. After 2 weeks, adult males which were ready to mate were put into the containers with the females. The first eggs were laid in early October. The cocoons were cut open, and the embryos removed from the cocoons were counted and divided into two groups. One group constituted a control sample which was stored under conditions optimal for the development of the species. The second group was exposed to a teratogenic factor in form of temperatures of 14 and 32 °C applied alternately at 12-hour intervals. After 10 days, i.e. after the first prosoma metameres appeared on the germ band, the eggs were transferred to an incubator with a constant room temperature of 23 °C. Larvae hatched about 20 days after the eggs were laid (the same as the control sample), and they were examined for developmental anomalies. Larvae with body malformations were reared in separate Petri dishes until they reached nymph stage II. Each developmental stage was identified on the basis of their external features in accordance with the nomenclature introduced by Vachon (1957). Individuals with body deformities (multiple anomalies and others) were examined for basic life functions and fixed in Bouin’s liquid prepared according to Zawistowski (Zawistowski 1986). After fixation, dehydration, and paraffin embedding, the preparations were cut into 7-µm-thick sections and coloured with Mayer’s haematoxylin and eosin.

## Results

Teratological experiments were carried out during six breeding seasons. A total of ca. 10,000 embryos were obtained, and half of the embryos were used as the control group. In the control group, no developmental anomalies were found, and the mortality rate was 4 %. In the teratological material, 674 individuals had miscellaneous malformations of appendages and structures of appendage origin (Table 1). The mortality rate of embryos in the experimental group was high, ca. 20 %.

**Table 1:** Observed cases of developmental anomalies in the prosoma in *Tegenaria atrica*

Kind of anomaly	Number of individuals
Oligomely	453
Heterosymely	64
Schistomely	61
Reduction in length	45
Polymely	23
Symely	15
Complex of anomalies	13
Total	674

In terms of morphological and anatomical structure, spiders with multiple anomalies were the most interesting because they are relatively rare, sometimes very complex, and the structure of the internal organs is still a problem that needs to be clarified. This group consisted of 13 individuals with several developmental anomalies, i.e. oligomely, heterosymely, polymely, and schistomely accompanied by significant reduction or malformations of distal leg parts. Basically, each specimen from this group was different. The histological analysis revealed changes in the central nervous system in the examined specimens. The changes concerned mainly the number of neuromeres. Spiders affected by polymely (eight out of 13 individuals) had additional ganglia corresponding to the number of additional appendages, whereas spiders affected by oligomely lacked a ganglion/ganglia corresponding to the missing leg/legs (five out of 13 individuals). The fusion of ganglia was found in all the three spiders with polymely of walking legs (with both simple and multiple anomalies). Nevertheless, walking legs on the polymelic side were not fused in any of the examined specimens. This paper focuses on the analysis of the central nervous system of four specimens out of the 13 affected by multiple anomalies and one specimen affected by pure polymely. Selected individuals showed the diversity of teratological changes observed during the experiment. Moreover, in three of them (spiders with polymely), fusion of ganglia was noticed for the first time.

### Case 1

**Fig. 1:**
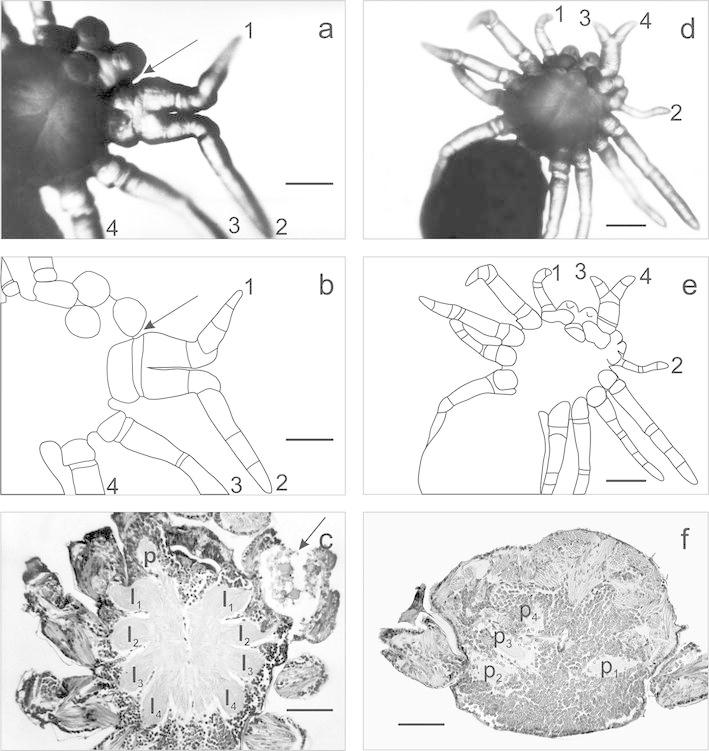
**a**, **b** *Tegenaria atrica* larva with oligomely of a pedipalp and partial heterosymely of walking legs 1 and 2 (*ventral view*): *1* and *2*—heterosymelic walking legs, the *arrow* indicates the location of a missing pedipalp, *scale bar* 0.14 mm, **c** frontal section through prosoma: p—pedipalp’s ganglion, *l*_1_–*l*_4_—ganglia of walking legs, the *arrow* indicates the fused midgut caeca, *scale bar* 0.09 mm, **d**, **e** larva of *Tegenaria atrica* with polymely and heterosymely of pedipalps (*ventral view*): *1* and *2*—correct pedipalps, *3* and *4*—polymelic pedipalps, *scale bar* 0.22 mm, **f** sagittal section through the anterior part of prosoma, *p*_1_—ganglion of pedipalp 1, *p*_2_—ganglion of pedipalp 2, *p*_3_ and *p*_4_—ganglia of fused polymelic pedipalps (3 and 4), *scale bar* 0.07 mm

The individual shown in Fig. 1a–c was affected by a combination of anomalies, i.e. by oligomely and partial heterosymely of the legs. The two anomalies affected the right side of the prosoma where a pedipalp was missing (oligomely), and the first and the second walking legs were partially fused (heterosymely). The fusion affected only the first, proximal podomeres of legs, i.e. coxae and trochanters, and part of the femurs. Other parts of the legs were not fused and moved independently of each other. The intersection of these legs grew much larger, while the free ends were of regular thickness and had all podomeres. The analysis of the central nervous system revealed changes in the number of neuromeres (Fig. 1c). One pedipalp ganglion was missing, i.e. the one corresponding to the missing appendage. However, the fusion of legs was not accompanied by the fusion of corresponding ganglia (l_1_,l _2_). The two ganglia were clearly visible and separated from each other. A nerve extended from each of them towards the corresponding part of the heterosymelic complex. The arrangement and the number of ganglia on the opposite sides of the prosoma were correct and corresponded to the number of appendages.

### Case 2

Another individual (Fig. 1d–f) had three pedipalps on the right side of the mouth. Apart from a regular pedipalp (2), there was a pair of pedipalps located next to each other that were partially fused (3 and 4). Apparently, the heterosymely caused the asymmetry of the prosoma. The fusion of heterosymelic pedipalps affected podomeres from coxae to patella (inclusive). As it was difficult to determine which of the four appendages were additional, it was assumed that the additional appendages were heterosymelic pedipalps (3 and 4). The analysis of the central nervous system based on the sagittal sections revealed changes in the side of the body affected by the anomaly. Three ganglia were found on this body side (Fig. 1f). Ganglia p_3_ and p_4_ innervated the heterosymelic complex (pedipalps 3 and 4), and the third ganglion (p_2_) was responsible for the innervation of pedipalp 2. Thus, it appears that partial fusion of pedipalps was not accompanied by the fusion of the corresponding ganglia. The examination of the nervous system based on the sagittal sections confirmed the initial assumption about additional (polymelic) pedipalps in the heterosymelic complex.

### Case 3

**Fig. 2:**
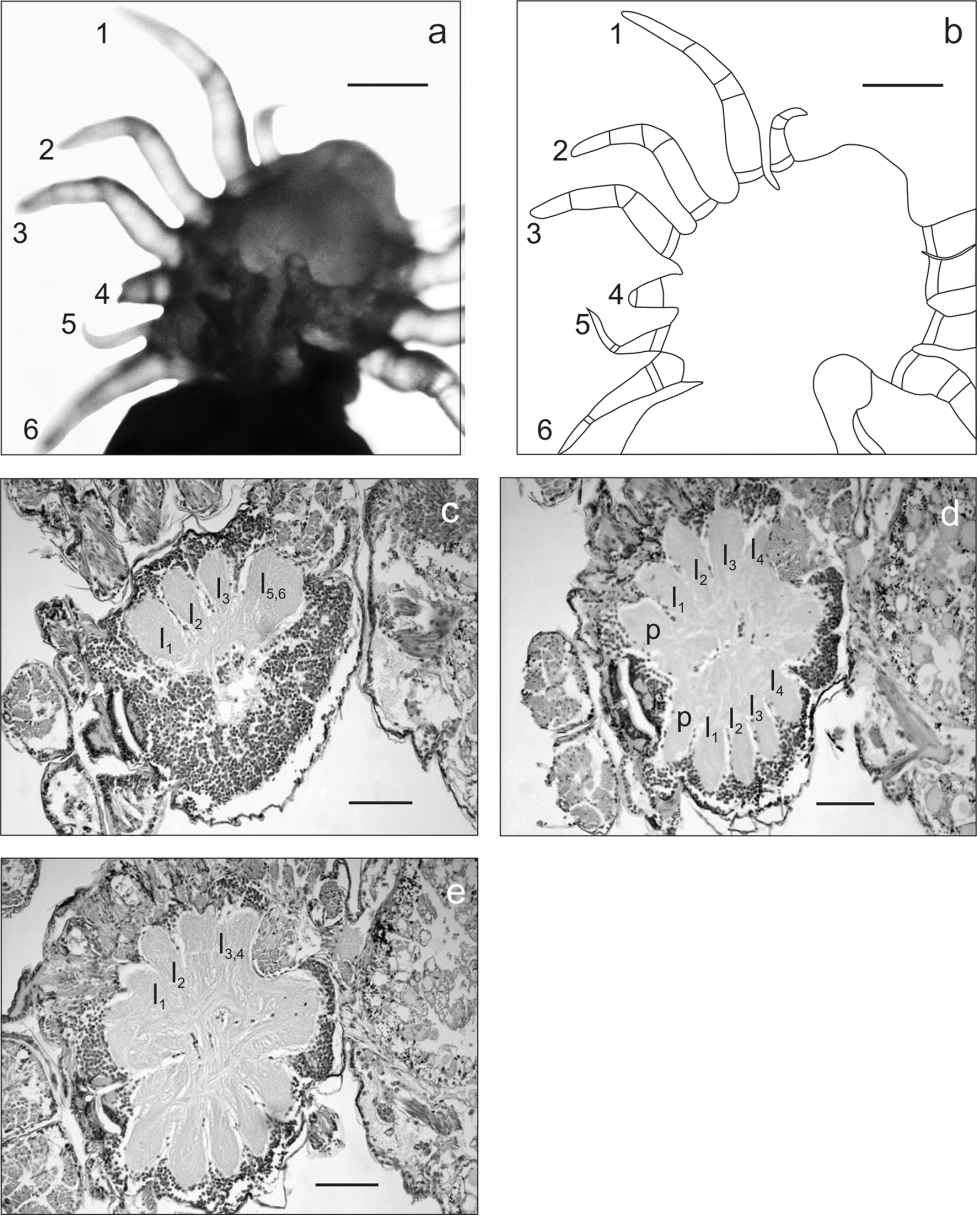
**a**, **b** *Tegenaria atrica* with the left-sided polymely of walking legs (*dorsal view*): *1*–*6*—walking legs, *scale bar* 0.23 mm, **c** frontal section through prosoma with ganglia of walking legs: *l*_1_, *l*_2_, *l*_3_, and *l*_5,6_, *scale bar* 0.12 mm; **d** frontal section through the suboesophageal neural mass: *p*—ganglia of pedipalps, *l*_1_, *l*_2_, *l*_3_, *l*_4_—ganglia of walking legs, *scale bar* 0.11 mm, **e** frontal section through the suboesophageal neural mass: *l*_3,4_—fused ganglia of walking legs 3 and 4, *scale bar* 0.11 mm

The specimen in Fig. 2 was affected by polymely of the walking appendages. In addition, the ends of the legs were shorter and deformed. The anomaly on the left side of the prosoma caused a serious malformation of the entire prosoma. On the right side of the prosoma, there were five walking appendages (1, 2, 3, 5, and 6) and one short stump (4). The first three walking legs (1, 2, and 3) were well developed and had a complete number of podomeres. Behind the legs, there was a three-podomere appendage in the form of a stump (4), and two other legs (5 and 6) slightly displaced towards the ventral side. One of them, the fifth leg, was much shorter compared to the normal walking leg. The last walking leg, the sixth one on this side of the prosoma, was also shorter. The joint surfaces of podomeres were distinguishable only up to the patella, while the other podomeres were constricted. On the opposite side of the prosoma, there were four normally developed walking legs. The analysis of CNS indicates major changes in the fused suboesophageal ganglia. Six pedal ganglia were found on the body side affected by the anomaly; their number corresponded to the number of legs. In none of the histological sections did all pedal ganglia occur together, which shows that neuromeres are displaced and located at different frontal planes. This particularly applies to the two last pedal ganglia (l_5,6_). They were clearly moved towards the ventral side, the same as the corresponding legs. Furthermore, the two last pedal ganglia were fused and formed one large, double neuromere (Fig. 2c—ganglion l_5,6_). This section shows three large ganglia (l_1_, l_2_, and l_3_), separated from each other with nerves diverging into the first three walking legs (1, 2, and 3). Furthermore, dorsal histological sections (Fig. 2d, e) did not show ganglion l_5_,_6_, but only one neural structure (l_4_). At the beginning, this neural section did not resemble (in size and shape) “the correct” pedal ganglia. The structure was contracted and relatively short, and its location indicated the association with the short stump (4) (Fig. 2d). On further, more dorsal sections, this ganglion was enlarged and fused with ganglion l_3_ (Fig. 2e). These two ganglia (l_3,4_) formed one large “double tongue” situated behind the ganglion of the second walking leg (l_2_). All properly developed ganglia of four walking appendages (l_1_–l_4_) were located on the other side of the prosoma.

### Case 4

**Fig. 3:**
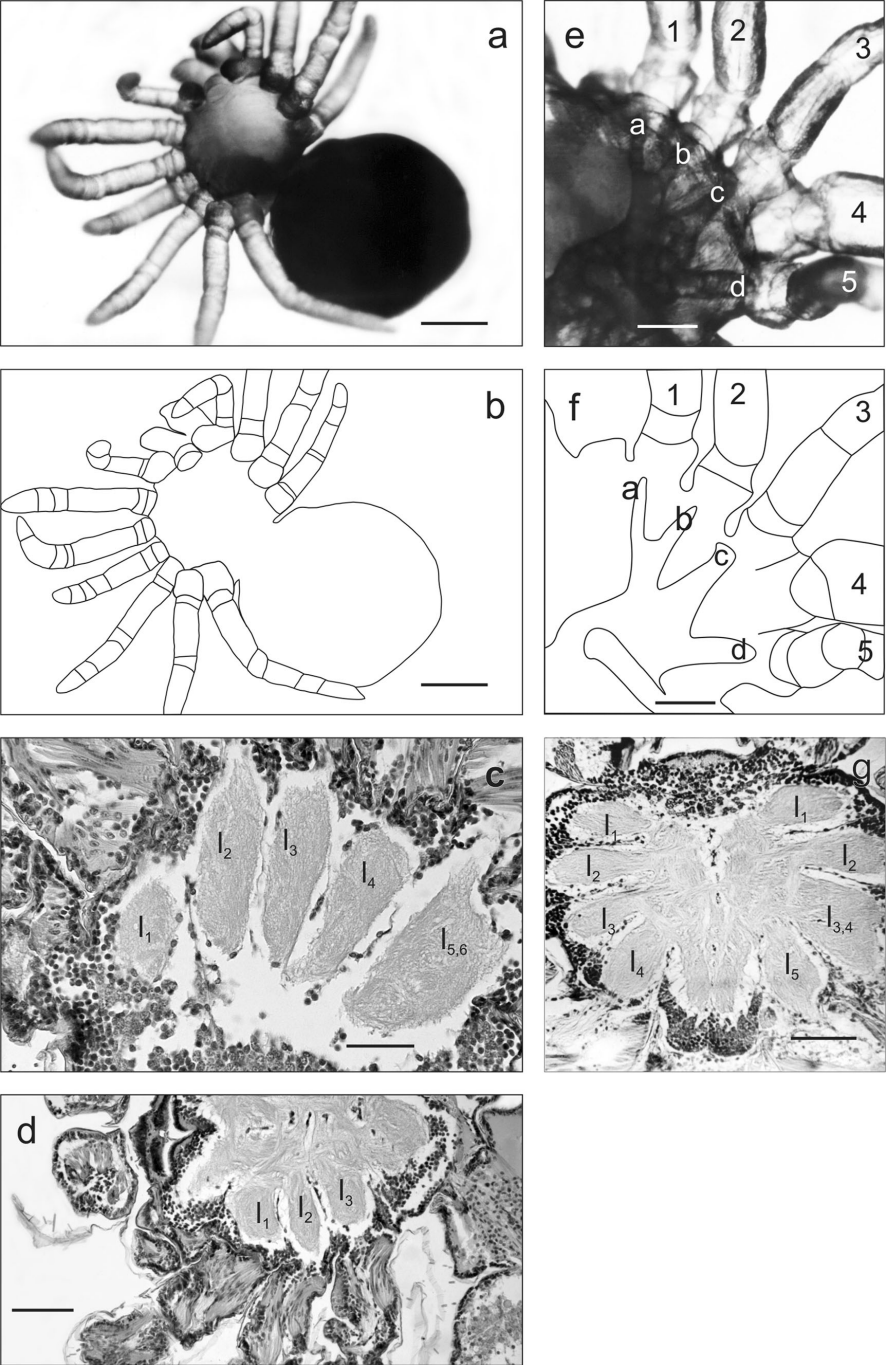
**a, b** *Tegenaria atrica* larva with polymely and oligomely of walking legs (*ventral view*), *scale bar* 0.22 mm, **c** frontal section through prosoma with ganglia of walking legs: *l*_1_, *l*_2_, *l*_3_, *l*_4_—ganglia of the first four walking legs, *l*_5,6_—fused ganglia of the two last walking legs, *scale bar* 0.05 mm, **d** section through the suboesophageal mass with three ganglia of walking legs (*l*_1_–*l*_3_) (oligomely), *scale bar* 0.11 mm, **e**, **f** larva of *Tegenaria atrica* with polymely of walking legs (*dorsal view*): *a*–*d*—midgut caeca, *1*–*5*—walking legs, *scale bar* 0.09 mm, **g** frontal section through suboesophageal neural mass: *l*_3,4_—two connected ganglia of leg 3 (normal) and 4 (polymelic)

The multiple anomaly found in another individual (Fig. 3a–d) consisted in simultaneous polymely and oligomely of the walking legs. Polymely occurred on the left side of the prosoma. There were two supernumerary legs. All walking legs on the left side of the body were of the same length, consisted of seven podomeres, and were arranged in a line on the edge of the prosoma. On the opposite side of the prosoma, there were only three walking legs (oligomely). These two deformities were responsible for considerable asymmetry of the prosoma. The analysis of the central nervous system revealed major changes in the fused suboesophageal ganglia. Six ganglia were found on the polymelic side of the body, with the fifth and the sixth ganglia fused to form one large, double ganglion (Fig. 3c, ganglia l_1_–l_5,6_). On the opposite sides of the body, there were only three ganglia corresponding to three walking legs (l_1_–l_3_) (Fig. 3d).

### Case 5

The fusion of the pedal ganglia was observed once again in this series of teratological studies, i.e. in the polymelic spider with one additional leg (Fig. 3e–g), located on the right side of the prosoma. It was the fourth (penultimate) leg distinguished by the lack of the corresponding midgut caeca and stiffness. On the left side of the prosoma, four pedal ganglia were found on frontal sections (l_1_–l_4_) and five ganglia on the opposite, polymelic side (Fig. 3g). The third and the fourth ganglia (l_3,4_) were fused and formed one large structure with two nerves extending towards the legs: the third (normal) and the fourth (polymelic) one.

## Discussion

Multiple biological and non-biological factors are considered as possible causes of morphological anomalies in invertebrates. Beside genetic factors (mutations of the germ or somatic cells), mechanical, physical, and chemical factors may influence embryos; parasites and predators may also cause anomalies. (Miličić et al. 2013). Among arthropods, morphological abnormalities are common in crustaceans (de Olivieira Dias 1999; Follesa et al. 2008; Feullassier et al. 2012) and in other animal taxa (Ćurčic et al. 1991; Reinert 1999; Mitić and Makarov 2007; Ferreira 2008, 2011, Mitić et al. 2011 Kozel and Novak 2013). Defects include deformities of the head, abdominal epimera, pleopods, telson, and uropods in crustaceans (Fernandez et al. 2011). Centipedes may have mispaired tergites, shrunk segments, variously deformed sclerites, bifurcated trunk, and defects of spiracles (Leśniewska et al. 2009). In insects, a majority of anomalies affect antennae, mandibles, legs, and the exoskeleton (Asiain and Márquez 2009). The analysis of anomalies in anthropods focuses mainly on morphological changes, while anatomical changes are rarely discussed. It is expected that significant modifications in the external structure lead to changes in the internal structure (e.g. in the nervous system). The research on oligomelic *T. atrica* spiders obtained by Jacuński et al. (2005) provides direct observation of this fact. They indicate that the absence of walking legs is always associated with the absence of the corresponding ganglia and leads to the reduction in the volume of the fused suboesophageal ganglia. However, the absence of some ganglia does not interrupt morphological and physiological continuity of the central nervous system nor does it impair the locomotion of the abnormal individuals. Jacuński et al. (2002) and Napiórkowska et al. (2006) studied other morphological defects of spiders through histological analysis. Polymelic individuals with one additional walking leg had an additional ganglion in the suboesophageal part of the nervous system. Similar results were obtained by Napiórkowska et al. (2010) who investigated the polymely of chelicerae. Although an additional ganglion was identified, there was no additional venom gland associated with the additional chelicerae. Jacuński et al. (2002) and Napiórkowska et al. (2013) performed morphological and anatomical analysis of spiders with partial and total heterosymely of appendages. Despite the fact that they were fused, no changes in the position or shape of the ganglia which innervated them were noticed. Only in two cases, both the appendages and the ganglia were fused. In all the remaining spiders, discussed by these authors, morphological changes were not reflected in the structure of the nervous system. The relationship between morphological defects and the structure of the central nervous system in *Tegenaria* affected by multiple anomalies was analysed in only one individual (Jacuński and Napiórkowska 2000). Three different types of anomalies, i.e. polymely, heterosymely, and schistomely, were diagnosed in this specimen. An additional ganglion and the fusion of two ganglia on the side affected by the anomaly, which would reflect the changes in the fused suboesophageal ganglia, were expected. Anatomical inspection confirmed the presence of an additional ganglion, which, however, was not fused with the ganglion of the schistomelic leg.

The analysis of the nervous system in spiders provides evidence that teratogenic factors may cause different malformations in its structure. In oligomely, this relationship is relatively simple: the absence of a walking leg is associated with the absence of the corresponding ganglion, and the number of missing neuromeres corresponds to the number of missing appendages. However, we have also encountered more complex cases, e.g. spiders affected by oligomely on one side of the prosoma and by polymely on the other. In these cases, additional ganglia were found only on the side affected by polymely (whereas on the side affected by oligomely the number of ganglia corresponded to the number of legs). It should be noted that the additional ganglia were well developed and separated from the other ganglia of walking legs and were either fused (when two additional legs were present), or the ganglion of the additional leg was fused with the ganglion of the leg formed during regular (undisturbed) ontogenesis. During our studies, fusion of ganglia in the case of spiders with polymely was found for the first time. However, the fusion of the ganglia on the polymelic side of the body did not involve the fusion of the legs. Moreover, in spiders affected by oligomely combined with polymely, the bilateral symmetry of the prosoma, as for the position of the ganglia forming the fused suboesophageal ganglia, was disturbed. Multiple anomalies visibly impaired vital body functions (e.g. locomotion). This was also observed in specimens with heterosymely of appendages (Napiórkowska et al. 2013). In individuals affected by heterosymely of feeding or walking legs combined with other anomalies (oligomely or polymely), heterosymely was observed only in the morphological structure. Contrary to expectations, the structure of the nervous system was unaffected: the ganglia were not fused.

The results of the research on spiders affected by both simple and multiple anomalies indicate that morphological deformities are not always reflected in the structure of the central nervous system. A direct relationship is observed only in oligomelic specimens: the absence of leg/legs entails the absence of corresponding ganglion/ganglia. However, in other types of simple deformities and multiple anomalies, there were a series of interactions during the formation of neuromeres, which could significantly complicate the anatomical image. As can be seen, morphological changes are not necessarily indicative of changes in the internal structure. Therefore, teratological studies should focus not only on the description and classification of defects but also on the anatomical analysis.

In conclusion, the central nervous system responds differently to teratogenic factors including temperature. The exposure of embryos to two alternating temperatures, both significantly deviating from the optimum, disturbs morphogenetic processes and results in a higher mortality rate and miscellaneous developmental defects. In our experiment, number and gravity of malformations were occasionally so serious that the embryos died before completing their development. Some individual went through the whole embryonic development but were unable to leave their egg capsules on their own and the experimenter’s attempts to help them were unsuccessful. These individuals were affected by complex, overlapping anomalies reflected in the body structure.
